# A PepFect14 analog improves non‐viral CRISPR delivery in primary human cells to facilitate genome editing and repair

**DOI:** 10.1002/btm2.70172

**Published:** 2026-08-28

**Authors:** Alex du Rand, Courtney Masterson, Daniel Verdon, Andrew Siow, Evert Loef, Rod Dunbar, Richard Kingston, Paul Harris, Hilary Sheppard

**Affiliations:** ^1^ School of Biological Sciences The University of Auckland Auckland New Zealand; ^2^ Maurice Wilkins Centre New Zealand

**Keywords:** cell penetrating peptides, CRISPR/Cas9, epidermolysis bullosa, gene editing, gene therapy, PepFect14, ribonucleoproteins

## Abstract

CRISPR‐based designer nucleases can facilitate genome engineering targeting almost any genomic locus. However, safe and efficient methods for delivering gene editors into primary human cells and tissues remain a central challenge. In this study, we employed a PepFect14 (PF14) analog, PF14‐K, to deliver high‐fidelity Cas9‐ribonucleoproteins and non‐viral repair templates into primary human skin cells to mediate gene editing and repair targeting genes underlying the group of genetic skin blistering disorders epidermolysis bullosa (EB). Peptide‐RNP nanoparticles enabled consistent gene editing of >70% in primary wild type fibroblasts and >50% in primary wild type keratinocytes. In more difficult‐to‐transfect primary EB skin cells, this strategy facilitated up to 68% exon deletion‐mediated reframing targeting *COL7A1* and 37% precise homology‐directed repair of a prevalent *LAMB3* mutation. Compared to electroporation, the gold standard for ex vivo delivery, PF14‐K enabled similar total yields of edited cells. Deliverable PF14‐K nanoparticles are highly cost‐effective, as they can be formed on the benchtop through a simple mix‐and‐incubate approach, with future potential to deliver base and prime editors.

**Translational Impact Statement**

Efficient and safe delivery of CRISPR gene editors into primary human skin cells remains a challenge. We describe a PepFect14 (PepFect14) cell penetrating peptide analog, PF14‐K, which enables efficient and minimally toxic delivery of Cas9‐ribonucleoproteins and non‐viral repair templates to facilitate genome engineering targeting genes underlying the group of genetic skin disorders epidermolysis bullosa. PF14‐K‐based nanoparticles hold promise for the delivery of diverse gene editors into primary human skin cells, and potentially the skin, for the future development of gene therapies.

**Abbreviations**
AAV: adeno‐associated virus
ANOVA: analysis of variance
cDNA: complementary DNA
CRISPR: clustered regularly interspaced short palindromic repeats
DAPI: 4′,6‐diamidino‐2‐phenylindole
DMEM: Dulbecco's modified eagle medium
DMSO: dimethyl sulfoxide
DPBS: Dulbecco's phosphate‐buffered saline
EtOH: ethanol
FBS: fetal bovine serum
FDA: food and drug administration
HBG: HEPEs buffered glucose
HIV: human immunodeficiency virus
HPRT: hypoxanthine‐guanine phosphoribosyltransferase
HSD: honestly significant difference
iPSCs: induced pluripotent stem cells
KGF: keratinocyte growth factor
MR: molar ratio
NEB: New England Biolabs
NIR: near‐infrared
PBS: phosphate‐buffered saline
PCR: polymerase chain reaction
RT: reverse transcription
RT‐PCR: reverse transcriptase polymerase chain reaction
SEM: standard error of the mean
SNP: single nucleotide polymorphism
TAE: tris‐acetate‐EDTA

## 1 INTRODUCTION

Genome engineering using designer nucleases has emerged as a valuable tool for fundamental and applied research. The CRISPR/Cas system is widely favored for its simplicity, versatility, and high efficiency. The most commonly used CRISPR/Cas system employs a Cas9 endonuclease derived from *Streptococcus pyogenes* (Sp) to generate double‐strand breaks (DSB) at target genomic sites containing a three nucleotide (5′‐NGG) protospacer adjacent motif (PAM) and defined by a 20 nucleotide chimeric single‐guide RNA (gRNA).^1^ DSBs are primarily repaired in a rapid fashion by error‐prone non‐homologous end joining (NHEJ), typically resulting in small insertions and deletions (INDELs) of <10 bp.^2^ Alternative end‐joining repair processes also contribute to mutagenic repair—for example, microhomology‐mediated end‐joining (MMEJ) facilitated by polymerase *θ*—which utilizes microhomologies on either side of the DSB, typically resulting in larger deletions. Precise, error‐free DSB repair is possible during the G2 and S phases of the cell cycle and is facilitated by the lower efficiency homology‐directed repair (HDR) pathways, which can be exploited for making precise sequence changes by providing a DNA repair template sharing homology with the target site.^2^

Scalable, efficient and safe delivery of CRISPR/Cas gene editors remains a central challenge for the translation of ex vivo and in vivo gene therapies.^3^ Viral delivery methods, particularly AAVs, enable highly efficient targeted gene editing, yet they are constrained by limited packaging capacity,^4^ the risk of concatemeric genomic insertions,^5^ activation of DNA damage response pathways,^6^ and potential genotoxicity from prolonged transgene expression.^4^ Chemical‐based delivery vectors including lipid nanoparticles (LNPs) represent a non‐viral alternative with several advantages, such as a high packaging capacity, low immunogenicity, and reduced susceptibility of the encapsulated cargo to nuclease degradation.^7^ However, the requirement for an expensive microfluidics device and synthesis of high‐quality mRNA can limit this method to well‐resourced labs. Nevertheless, LNPs are currently a leading delivery method in the gene therapy field and have already demonstrated a high degree of success in the clinic for in vivo Cas9 delivery.^8^, ^9^

Physical delivery methods, namely electroporation, have dominated the ex vivo CRISPR/Cas landscape for preclinical gene therapy studies^10^ and clinical trials,^11^ owing to their efficient nuclear delivery capacity.^12^, ^13^ Compared to viral vectors or delivery of mRNA which rely on protein translation, electroporation of ribonucleoproteins (RNPs) offers improved safety due to their short intracellular half‐life. In 2023, electroporation was employed in the first FDA‐approved CRISPR gene therapy—Casgevy™—for treating sickle cell disease.^14^ However, despite its success, electroporation can introduce a substantial source of cytotoxicity^15^ and cause widespread transcriptomic alterations.^16^ Additionally, the need for specialized hardware and buffers, combined with limited applicability for in vivo applications, presents additional hurdles.

Cell penetrating peptides (CPPs) are a diverse group of short (<30 amino acids) peptides capable of translocating cell membranes and represent a promising alternative CRISPR delivery method.^17^ Unlike viral delivery or electroporation, delivery with CPPs can be hindered by endosomal entrapment if cargo is transported via endocytosis. Following endosomal escape, CRISPR enzymes also require nuclear import to facilitate gene editing, which is facilitated by a nuclear localization signal. While chemical methods can be used to conjugate CPPs to cargo for facilitating delivery,^18^, ^19^ an alternative approach involves mixing and incubating cationic CPPs with RNPs to form non‐covalent complexes which can be applied to cells in culture to facilitate intracellular delivery and gene editing.^16^, ^20^, ^21^, ^22^ This “mix‐and‐incubate” strategy has proven to be minimally toxic and simple as CPPs can be commercially synthesized at low cost. Foss and colleagues employed this strategy using the amphiphilic A5K peptide and achieved Cas9‐RNP‐mediated gene editing rates of up to 67% in primary human immune cells with minimal toxicity.^16^ However, delivery of non‐viral DNA repair templates was inefficient, highlighting the challenge of delivering DNA templates into the nucleus using CPPs, prompting the use of AAV6 vectors. The “mix‐and‐incubate” strategy has also been investigated in adherent human cells but has typically been performed in cell lines which are highly permissible to transfection, with Cas9 often targeted to reporter constructs which are usually more accessible than endogenous genes.^20^, ^21^, ^22^, ^23^ For example, the amphiphilic LAH5 CPP was used to facilitate HDR of up to 20% in HEK293T stoplight reporter cells,^22^ while another group reported <1% HDR using the RALA CPP when targeting the Rosa26 safe harbor site.^20^ In a separate study, Gustafsson et al. repurposed the amphiphilic peptide PepFect14 (PF14), containing an N‐terminal stearic acid moiety to promote endosome escape, to deliver Cas9‐RNPs into HEK293T cells, achieving ~50% gene editing targeting a stably integrated stoplight reporter construct, or 33% when targeting the endogenous HPRT gene.^21^ Additionally, the capacity of PF14 to deliver nucleic acids into primary human adherent cells has previously been demonstrated.^24^ Collectively, these results led us to investigate whether PF14 could be used for CRISPR delivery in primary human skin cells for genome editing and repair.

In this study, we employed a PF14 variant (PF14‐K) containing an additional C‐terminal lysine. PF14‐K was used to deliver high‐fidelity Cas9‐RNPs and short single‐stranded HDR templates (HDRT) into primary human keratinocytes and dermal fibroblasts derived from healthy donors or people with epidermolysis bullosa (EB). EB is a group of rare monogenetic skin blistering disorders characterized by pathogenic mutations in one of ~20 genes encoding critical structural proteins which enable stable attachment of the dermal and epidermal skin layers.^25^ Junctional EB (JEB) is a severe subtype primarily caused by mutations in the *LAMB3* gene, while recessive dystrophic EB (RDEB) represents a severe monogenic subtype caused by mutations in the *COL7A1* gene.^25^ Several gene editors based on the prototypic SpCas9 nuclease system have been investigated pre‐clinically to address these mutations, mostly using electroporation for delivery in an ex vivo setting.^10^ Our group and others have shown that Cas9 nuclease strategies can be efficiently employed to precisely correct pathogenic variants^26^, ^27^, ^28^, ^29^ or to delete mutation‐harboring *COL7A1* exons for reading frame restoration.^30^, ^31^, ^32^

Taken together, we demonstrate efficient Cas9‐RNP gene editing facilitated by PF14‐K in primary human skin cells with improved viability compared to electroporation, resulting in similar yields of edited cells. Gene editing with PF14‐K enabled efficient deletion of faulty *COL7A1* exons and precise HDR‐mediated correction of a prevalent *LAMB3* mutation using non‐viral DNA templates. CRISPR‐mediated HDR and exon skipping to repair an endogenous gene has not been previously demonstrated using a non‐viral CPP strategy in primary human cells. This simple and inexpensive method has the potential to be applied to deliver other gene editors and could be explored for in vivo gene editing in the skin for EB.

## 2 RESULTS

### 2.1 Cas9‐RNP delivery strategy facilitated by the cell penetrating peptide PepFect14 in primary human skin cells

We investigated whether PF14—previously demonstrated to deliver Cas9‐RNPs into HEK293T cells^21^—could be used for the same purpose in primary human skin cells for genome editing and repair. We employed a similar mix‐and‐incubate strategy whereby preformed high‐fidelity Cas9‐RNPs are incubated with a molar excess of PF14 in a complexation buffer containing the crowding reagent polyethylene glycol‐polyvinyl alcohol (PEG‐PVA) to enhance complexation efficiency (Figure 1a, Figure S1).^21^ Cas9‐RNP/PF14 nanocomplexes are then applied to primary skin cells in culture for 24 h, and genomic DNA extracted after a further 2 days to assess genome editing efficiency using Sanger or Nanopore sequencing (Figure 1a). Ornithine residues within PF14 enhance peptide stability against serum proteases, enabling transfection in full‐serum medium (Figure 1b).

**FIGURE 1:**
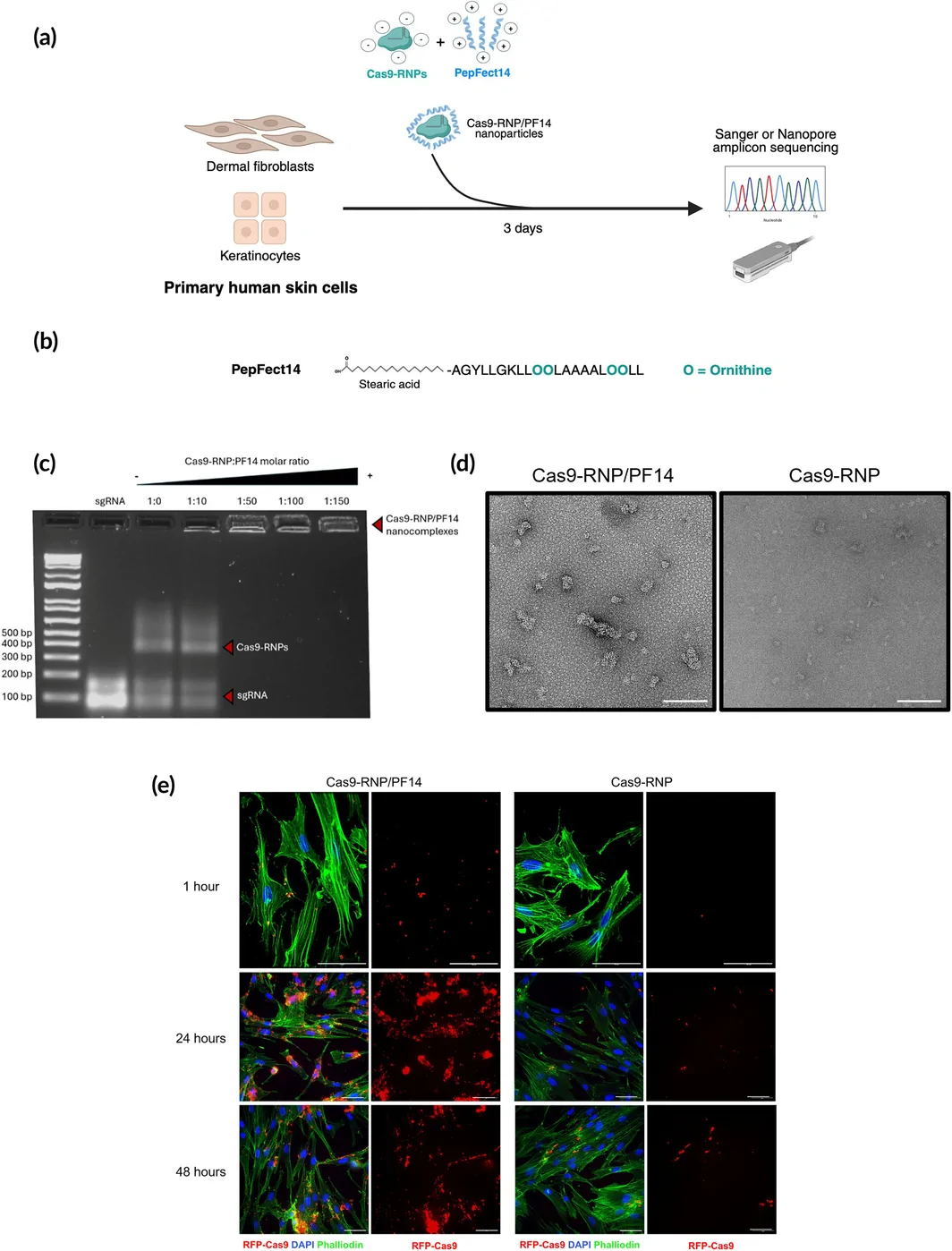
Overview of Cas9‐ribonucleoproteins (Cas9‐RNP) delivery strategy facilitated by the cell penetrating peptide PepFect14 (PF14) in primary human skin cells. (a) Schematic of “mix‐and‐incubate” Cas9‐RNP delivery workflow facilitated by PF14. (b) PF14 peptide sequence, containing an N‐terminal stearic acid and the non‐natural amino acid ornithine introduced to reduce degradation by serum proteases. (c) Electrophoretic mobility shift assay of Cas9‐RNP/PF14 nanocomplexes at varying molar ratios. sgRNA or Cas9‐RNP without PF14 were included as controls. (d) Representative transmission electron microscopy images of Cas9‐RNPs or Cas9‐RNP/PF14 nanocomplexes. Scale bars = 80 μm. (e) Representative confocal microscopy images of primary dermal fibroblasts after different timepoints following treatment with Cas9‐RNP/PF14 complexes or Cas9‐RNPs, using a red‐fluorescently tagged Cas9 (RFP‐Cas9). DAPI was used as a nuclear stain and phalloidin as a cytoplasmic stain. Scale bars = 100 μm.

PF14 has a high isoelectric point and thus carries a strong positive charge at neutral pH, enabling it to electrostatically interact and form complexes with negatively charged cargo including nucleic acids or the gRNA component of Cas9‐RNPs.^33^ To investigate nanocomplex formation, we assessed the mobility of pre‐incubated Cas9‐RNPs with increasing molar ratios of PF14 using gel electrophoresis (Figure 1c). As expected, gRNA or preformed Cas9‐RNPs without PF14 showed high electrophoretic mobility given their strong negative charge at the pH (8.3) of the TAE buffer used in the assay. However, the addition of positively charged PF14, even at the lowest molar ratio, appeared to reduce the electrophoretic mobility of the Cas9‐RNPs, as evidenced by the retention of some fluorescent signal within the loading well. Complete loss of electrophoretic mobility was observed for all higher molar ratios, indicative of nanocomplex formation and Cas9‐RNP charge neutralization by PF14 (Figure 1c). Nanocomplex formation was corroborated by transmission electron microscopy (TEM) which revealed a substantially larger size of Cas9‐RNP/PF14 nanocomplexes formed at a 1:100 molar ratio (determined from screening in Figure 2a) compared to Cas9‐RNPs alone (Figure 1d).

We next assessed the cellular uptake of Cas9‐RNP/PF14 nanocomplexes. Preformed Cas9‐RNPs containing Cas9 conjugated to red‐fluorescent protein (RFP‐Cas9) were incubated with PF14 at a 1:100 molar ratio. Nanocomplexes were added to the medium of primary wild type neonatal dermal fibroblasts in culture for one, 24, or 48 h, after which the cells were fixed and analyzed using confocal microscopy (Figure 1e). In the absence of peptide, dim intracellular RFP fluorescence was detected across all timepoints, indicating low levels of passive Cas9‐RNP uptake (Figure 1e). Upon addition of peptide to Cas9‐RNPs, a large increase in intracellular RFP fluorescence was observed across all timepoints. After 1 h, RFP fluorescence was mostly punctate, likely reflecting Cas9‐RNP/PF14 complexes trapped within endosomes. A substantial increase in RFP signal was detected after 24 h and was characterized by more diffuse cytoplasmic staining, as well as nuclear staining. After 48 h, a slight reduction in RFP fluorescence was observed, suggesting RFP‐Cas9 degradation (Figure 1e).

### 2.2 Delivery of Cas9‐RNPs facilitated by PF14 at a 1:100 molar ratio is associated with improved cell viability compared to electroporation

We next performed a titration to determine the optimal Cas9‐RNP dose and Cas9‐RNP to PF14 molar ratio in primary wild type keratinocytes and dermal fibroblasts. Experiments were performed in 96‐well plates, with three Cas9‐RNP concentrations evaluated (10, 13.5, and 17 nM, empirically determined from initial screening) corresponding to 160, 214, and 270 ng of Cas9 per well, respectively. For each Cas9 dose, four Cas9‐RNP to PF14 molar ratios were tested ranging from 1:50 to 1:200 (Figure 2a). Viability was measured after 24 h using the AlamarBlue assay, which quantifies the metabolic activity of cells in culture by measuring the reduction of a blue indicator dye resazurin into the highly fluorescent compound resorufin. Gene editing efficiency was determined using Sanger sequencing. A gRNA targeting intron 67 of the *COL7A1* gene was used to avoid altering a coding sequence which could have confounding effects on cell viability (Table S1). We compared gene editing efficiencies and cell viability to electroporation‐mediated delivery, using the standard pulse program DT‐130 for dermal fibroblasts (recommended by Lonza), and CM‐137 for keratinocytes which is standard in our laboratory.^26^, ^28^, ^31^ Our previous optimization found that CM‐137 is a more efficient pulse program than DS‐138 (which is recommended by Lonza) with comparable cell viability.

**FIGURE 2:**
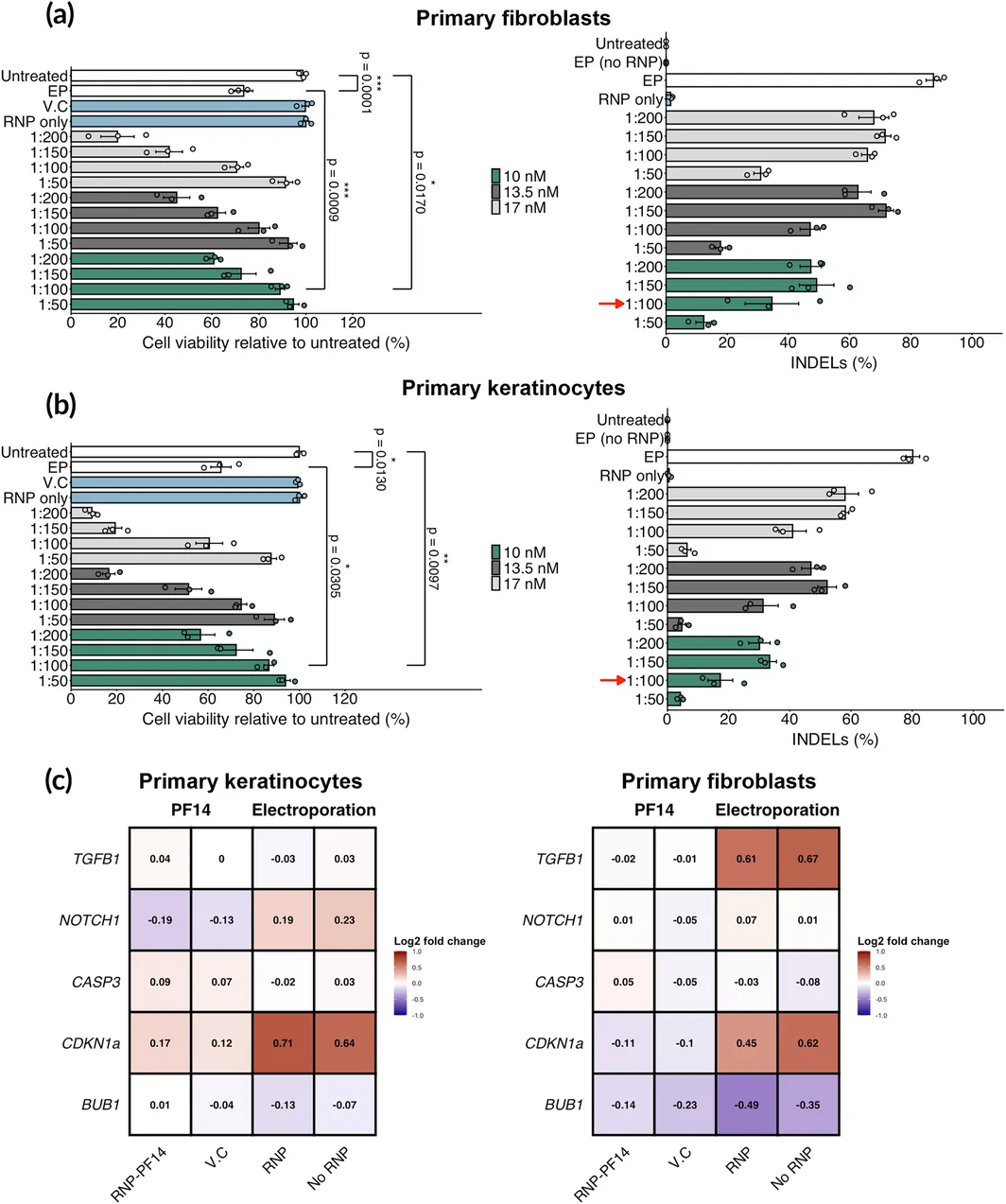
Evaluation of different Cas9‐ribonucleoproteins (Cas9‐RNP) and PepFect14 (PF14) concentrations on cell viability and gene editing in primary human skin cells compared to electroporation. Titration of Cas9‐RNP concentration and Cas9‐RNP to PF14 molar ratio is depicted for primary human dermal fibroblasts (a) and primary human keratinocytes (b), with cell viability (relative to untreated cells) shown on the left and gene editing efficiencies on the right. Four Cas9‐RNP to PF14 molar ratios (1:50 to 1:200) were evaluated across three Cas9‐RNP concentrations compared to electroporation (EP). For viability analysis, vehicle control (V.C) and Cas9‐RNP without PF14 (RNP only, blue bars) were included as controls for peptide‐mediated delivery. A red arrow depicts the condition chosen for subsequent optimization. Three biological replicates performed (*n* = 5). Bars represent the mean. Error bars represent the SEM *p*‐Values were generated from one‐way ANOVA with post hoc Tukey HSD; *< 0.05, **< 0.01, and ***< 0.001. (c) Absolute quantification of gene expression in primary human keratinocytes (left) or dermal fibroblasts (right) 24 h after gene editing facilitated by PF14 or electroporation. Gene expression is represented as the log2 fold change relative to untreated cells and represents the mean of two individual experiments.

A dose‐dependent reduction in cell viability was observed with increasing Cas9‐RNP concentrations and Cas9‐RNP to PF14 molar ratios (Figure 2a,b). Peptide concentrations exceeding ~1.5 μM were associated with a reduction in viability of typically less than 70%, with the highest dose (17 nM Cas9‐RNP, 3.4 μM PF14, 1:200 molar ratio) resulting in an average viability of 19% and 9% viability in dermal fibroblasts and keratinocytes, respectively (Figure 2a,b). Conversely, a dose‐dependent increase in gene editing was observed with increasing Cas9‐RNP concentrations, with the highest gene editing of 75.3% and 70.4% achieved with 17 nM Cas9‐RNP at higher molar ratios in dermal fibroblasts and keratinocytes, respectively (Figure 2a,b). Across both cell types, the 1:100 molar ratio using the lowest Cas9‐RNP dose of 10 nM was associated with an average cell viability of 90% compared to 74% with electroporation in dermal fibroblasts (*n* = 3, *p* = 0.0009), and 86% compared to 66% in keratinocytes (*n* = 3, *p* = 0.03) (Figure 2a,b). Under this condition, PF14 facilitated gene editing efficiencies of up to 50.3% (average = 35.2%, *n* = 3) compared to 90.9% (average = 89.7%, *n* = 3) by electroporation in dermal fibroblasts, and 25.2% (average = 18.6%, *n* = 3) in keratinocytes compared to 84.5% (average = 81.7%, *n* = 3) by electroporation (Figure 2a,b). Although editing rates were lower compared to electroporation, we pursued this condition for subsequent optimization since 1 μM is a routinely used concentration,^24^, ^33^ and higher peptide concentrations resulted in a substantial drop in cell viability (<70%) (Figure 2a,b).

Using the optimized conditions described above (10 nM Cas9‐RNP, 1:100 molar ratio), we assessed the expression of five genes–*TGFB1*, *NOTCH1*, *BUB1*, *CASP3*, *CDKN1A*—following gene editing facilitated by PF14 or electroporation. These genes are involved in a variety of fundamental cellular processes including apoptosis (*CASP3*, *TGFB1*), proliferation and differentiation (*TGFB1*, *NOTCH1*), and cell cycle kinetics (*CDKN1A*, *BUB1*). To ensure sufficient cell numbers for downstream analysis, these experiments were proportionally scaled up to a 12‐well plate. mRNA was extracted from primary keratinocytes and fibroblasts 24 h after treatment and gene expression quantified using droplet digital PCR (ddPCR) (Figure 2c, Table S2). An increase in the expression of *CDKN1A* was observed in both cell types following electroporation, indicative of cell cycle arrest,^34^ with a concomitant decrease in *BUB1*–a master regulator of mitotic spindle assembly—also observed in fibroblasts.^35^ In addition, elevated *TGFB1* expression was observed following electroporation in fibroblasts, a hallmark of fibroblast activation which can induce differentiation into myofibroblasts.^36^ Collectively, these preliminary data indicate that electroporation may alter key cellular pathways to a greater extent than PF14.

### 2.3 Efficient peptide‐mediated Cas9‐RNP gene editing in primary human skin cells

Encouraged by the improved cell viability observed at the 1:100 Cas9‐RNP to PF14 molar ratio (10 nM Cas9‐RNP) compared to electroporation, we hypothesized that gene editing rates could be improved by optimizing factors influencing cellular uptake. To this end, we engineered a PF14 variant containing a C‐terminal bromoacetic acid moiety (Figure 3a). We reasoned that the electrophilic carbon within the bromoacetic acid could potentially facilitate more efficient complexation by reacting with the nucleophilic functional groups of the lysine residues on the Cas9 surface. A lysine residue containing a reactive amine group was added to the C‐terminal of PF14 to facilitate attachment of the bromoacetic acid. To ensure a fair comparison with the original PF14, we engineered an additional PF14 variant containing the same C‐terminal lysine but without the bromoacetic moiety (hereafter referred to as PF14‐K) (Figure 3a). Interestingly, PF14 with the bromoacetic acid demonstrated similar complexation with Cas9‐RNPs compared to standard PF14 using an electrophoretic mobility shift assay (data not shown) but did not result in any detectable gene editing. However, the PF14‐K variant resulted in subtle but consistent improvements in gene editing across all three tested gRNAs (Figure 3b, Table S1). Encouraged by this improvement, we engineered a final variant with the N‐terminal stearic acid of PF14‐K replaced with oleic acid (PF14‐KO), a modification previously demonstrated to enhance Cas9‐RNP delivery and gene editing with the LAH5 amphiphilic CPP.^37^ However, this modification resulted in a loss of efficiency compared to PF14 and PF14‐K across all tested gRNAs (Figure 3b).

**FIGURE 3:**
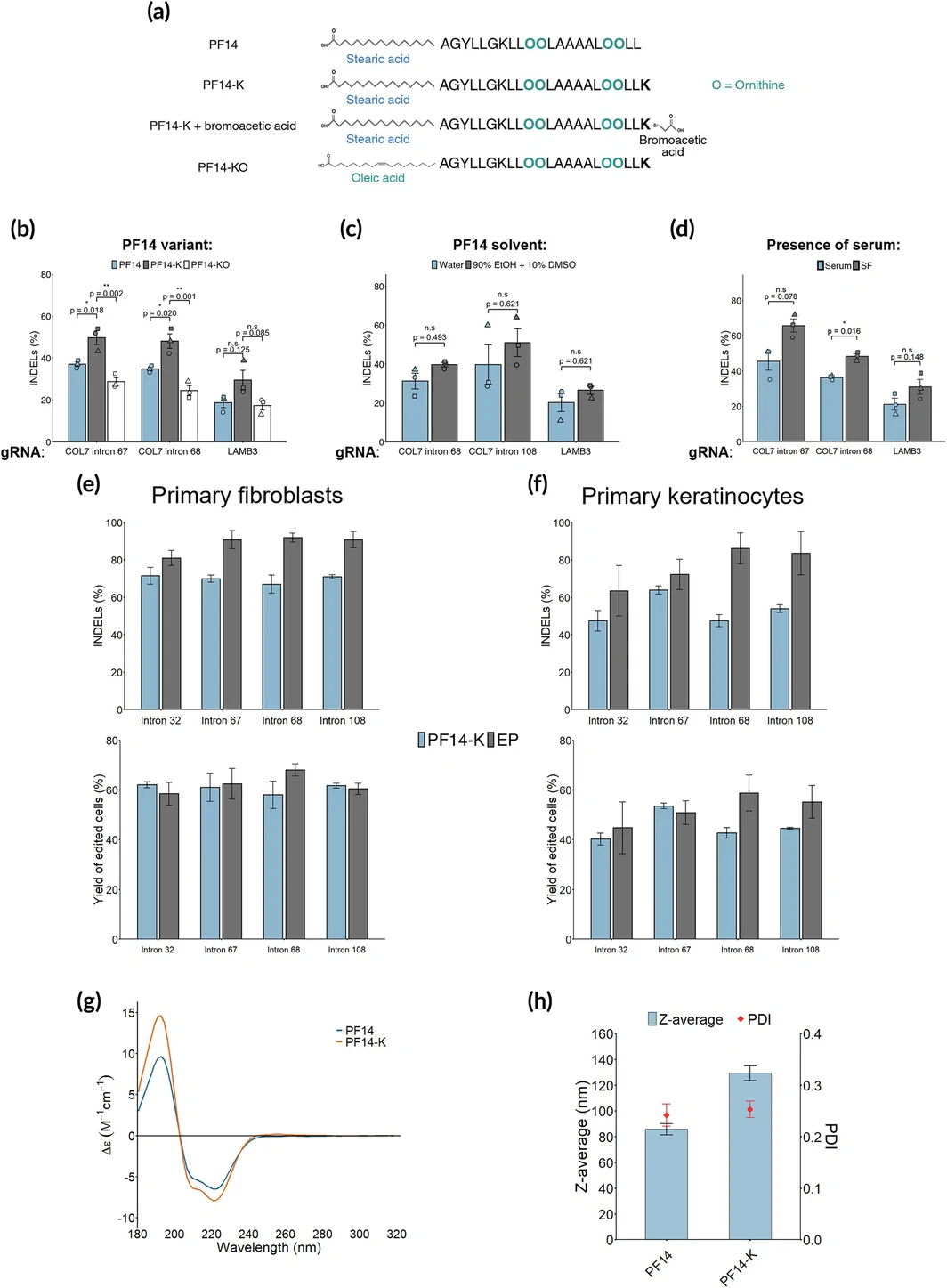
Optimisation of peptide‐mediated Cas9‐ribonucleoproteins (Cas9‐RNP) gene editing in primary human skin cells. (a) Schematic of different PepFect14 (PF14) analogs evaluated for Cas9‐RNP delivery in primary human skin cells. Comparison of gene editing efficiencies facilitated by (b) three PF14 analogs, (c) PF14 dissolved in two different solvents, or (d) treatment with PF14/Cas9‐RNP in serum versus serum‐free medium in primary dermal fibroblasts. Three biological replicates performed. Bars represent the mean. Error bars represent the SEM Biological replicates are represented as shapes from matched experiments. *p*‐Values were generated from one‐way ANOVA with post hoc Tukey HSD for (b) and Welch's *t*‐test with Holm–Šidák correction for multiple comparisons for (c) and (d). *< 0.05, **< 0.01, and ns (no significance). Optimized Cas9‐RNP gene editing conditions facilitated by PF14‐K in primary dermal fibroblasts (e) and primary keratinocytes (f) compared to electroporation (EP). Gene editing efficiencies (top) and cell viability relative to untreated cells (bottom) are shown for both cell types. Bars represent the mean of two biological replicates. Error bars represent the SEM (g) CD spectra of PF14 and PF14‐K represented as Δ*ε* per mol of peptide bond. (h) Dynamic light scattering analysis of PF14 and PF14‐K depicting the *Z*‐average hydrodynamic diameter (nm) and polydispersity index (PDI). Three measurements were taken per sample. Error bars represent the SEM of two experiments.

Next, we investigated whether using an organic solvent for dissolving PF14 rather than water could enhance gene editing. Biswas et al. demonstrated that using 90% EtOH + 10% DMSO as a solvent for PF14 reduced the formation of large aggregates with or without nucleic acid cargo, leading to more monodisperse solutions and enabling subtle enhancements in the cellular delivery of splice‐switching oligonucleotides.^38^ Similarly, we observed subtle increases in gene editing across three gRNAs when using this same solvent compared to water. However, while these differences did not reach statistical significance, consistent improvements in gene editing were observed across the three individual experiments (datapoints from matched experiments are indicated in Figure 3b–d).

Several non‐natural ornithine amino acid residues were introduced into the design of PF14 sequence to enhance its stability in full‐serum medium.^33^ However, we observed consistent improvements in gene editing when transfection was performed in serum‐free conditions, with the increases for two out of three tested gRNAs reaching statistical significance (Figure 3d).

Based on the above optimization, we proceeded with serum‐free transfection of Cas9‐RNP/peptide nanocomplexes, using PF14‐K dissolved in 90% EtOH + 10% DMSO. Under these conditions, gene editing efficiencies of 70%–75% and 50%–60% were routinely achieved in primary fibroblasts and keratinocytes, respectively (Figure 3e,f). Although electroporation was more efficient (>80%–90% in fibroblasts and 60–90% in keratinocytes), gene editing with PF14‐K was typically associated with >80%–90% cell viability compared to ~70% with electroporation, resulting in similar edited total cell yields (Figures 3e,f and S2).

To more fully understand the mechanism behind the observed improvement in gene editing facilitated by PF14‐K, we performed circular dichroism (CD) spectroscopy to assess peptide secondary structure. Previous research has demonstrated that PF14 adopts an α‐helical structure in aqueous solution which is essential for its delivery function.^39^ A small increase in α‐helical content was observed for PF14‐K compared to PF14, as evidenced by the increased intensity of the positive band at 190 nm and the negative bands at 208 and 222 nm (Figure 3g). However, there was no observable difference in thermal stability of the α‐helix (Figure S3). Dynamic light scattering (DLS) measurements revealed larger peptide/Cas9‐RNP particle sizes with PF14‐K compared to PF14 (130 and 90 nm, respectively), with similar polydispersity index (PDI) measurements (Figure 3h). Together, these data suggest a potentially subtle difference in α‐helical content between peptide analogs, and a slightly larger nanoparticle size when complexed with Cas9‐RNPs.

### 2.4 PF14‐K facilitates efficient deletion of *COL7A1* exons in primary keratinocytes and dermal fibroblasts

The excision of faulty *COL7A1* exons using dual Cas9‐RNPs has previously been demonstrated by our group and others as a potential gene editing strategy for RDEB^30^, ^31^, ^32^ and has consistently enabled highly efficient rates of targeted gene editing. Thus, we next assessed the ability of PF14‐K to deliver dual Cas9‐RNPs to facilitate excision of *COL7A1* exons 31, 68, and 109 in primary wild type dermal fibroblasts using previously published gRNAs (Figure 4a, Table S1).^31^ Individual gRNAs within each pair were used at a final concentration of 5 nM to maintain the final Cas9‐RNP concentration at the optimized 10 nM concentration. Genomic DNA was extracted from gene‐edited cells and polymerase chain reaction (PCR) used to amplify regions (<1000 bp) spanning the target exons (Table S2). Lower molecular weight agarose gel bands indicated excision of all three exons facilitated by PF14‐K, albeit lower compared to electroporation, ranging from 8% to 42% depending on the targeted exon (Figure 4b).

**FIGURE 4:**
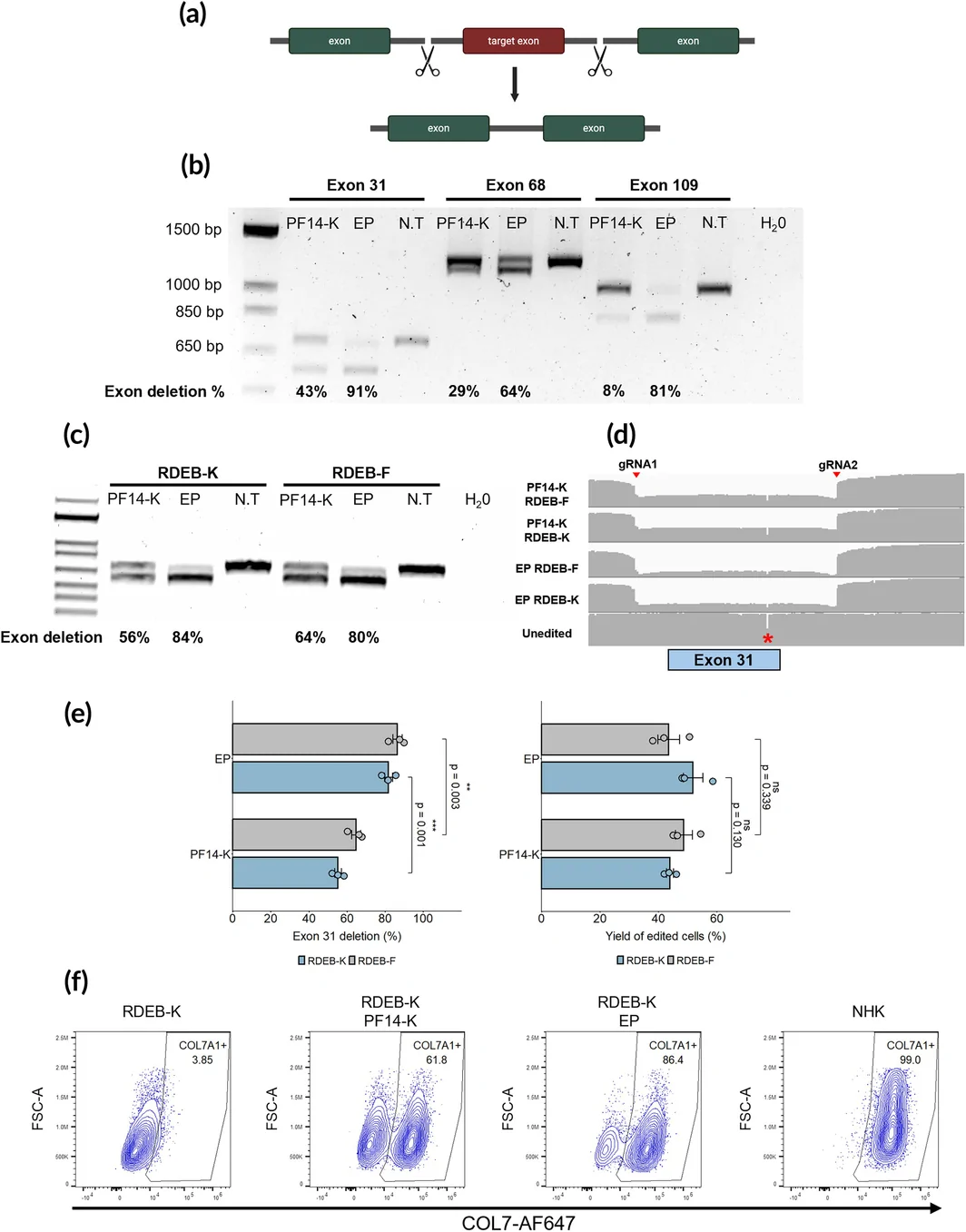
Dual Cas9‐ribonucleoproteins (Cas9‐RNP) exon skipping facilitated by PepFect14 variant (PF14‐K) targeting *COL7A1* in primary skin cells derived from healthy or recessive dystrophic electroporation (RDEB) donors. (a) Schematic diagram of dual Cas9‐RNP gene editing strategy targeting *COL7A1*. (b) Agarose gel assessment of DNA amplicons following dual Cas9‐RNP exon skipping in primary dermal fibroblasts derived from healthy donors targeting three *COL7A1* exons facilitated by PF14‐K or electroporation (EP). Lower molecular weight bands correspond to alleles with deletion of the targeted exon. Exon deletion efficiencies are estimated based on densitometry. Water (H_2_0) was run as a loading control. N.T, no treatment. (c) Agarose gel assessment of DNA amplicons spanning exon 31 following dual Cas9‐RNP exon skipping facilitated by PF14‐K or EP in primary RDEB keratinocytes (RDEB‐K) and RDEB fibroblasts (RDEB‐F). Exon deletion efficiencies are estimated based on densitometry. Water (H_2_0) was run as a loading control. N.T., no treatment. (d) Histograms depicting read depth of Nanopore amplicon sequencing reads aligned to the human reference genome viewed in interactive genomics viewer. The positions of the gRNAs and exon 31 are shown. The heterozygous mutation site is depicted with a red asterisk. A drop off in coverage represents deletion of the target exon. (e) Exon deletion efficiencies following dual Cas9‐RNP exon skipping facilitated by PF14‐K compared to EP in primary RDEB keratinocytes or fibroblasts (left), with edited cell yields shown on the right. Three biological replicates performed. Bars represent the mean. Error bars represent the SEM *p*‐Values were generated using the Welch's *t*‐test with Holm–Šidák correction for multiple comparisons. **<0.01, ***<0.001, ns = no significance. (f) Flow cytometric analysis of COL7 in RDEB‐K following dual Cas9‐RNP‐mediated exon 31 deletion facilitated by PF14‐K or EP. Data are representative of two individual experiments. NHK, normal human keratinocytes.

Next, we evaluated the capacity of PF14‐K to excise exon 31 containing a heterozygous 1 bp deletion (c.3840del) in primary RDEB keratinocytes (RDEB‐Ks) and RDEB fibroblasts (RDEB‐Fs). Again, lower molecular weight bands indicated exon deletion in both cell types, with densitometric analysis indicating efficiencies exceeding those achieved in wild type cells (56% for RDEB‐K and 64% for RDEB‐F) (Figure 4c). Nanopore‐based amplicon sequencing revealed exon 31 deletion efficiencies of up to 58.4% in RDEB‐Ks (average = 55.2%, *n* = 3) and 67.8% in RDEB‐Fs (average = 64.7%, *n* = 3) (Figure 4d,e). Efficiencies of up to 85.5% and 89.9% were achieved in RDEB‐Ks and RDEB‐Fs by electroporation (average = 81.7% and 86.3% for RDEB‐K and RDEB‐F, *n* = 3), respectively, but this was associated with a substantial source of cytotoxicity (average = 50.7% viability relative to untreated for RDEB‐Fs and 63.3% for RDEB‐Ks, *n* = 3, Figure S4), reflecting the challenge of working with difficult‐to‐transfect primary RDEB cells with poor proliferative capacity (Figure 4e). Cell viability with PF14‐K was ~80% in primary RDEB cells, lower than observed in wild type primary skin cells, but enabled similar total edited cell yields compared to electroporation (Figures 4e and S4). Flow cytometric analysis corroborated the sequencing data, demonstrating COL7 restoration of up to 61% in RDEB‐Ks following PF14‐K treatment, compared to 86.4% by electroporation (Figures 4f, S5, and S6). Collectively, these data highlight the potential of PF14‐K as a promising alternative delivery method for primary cells which are refractory to electroporation.

### 2.5 Therapeutic HDR‐mediated correction of a prevalent *LAMB3* mutation facilitated by PF14‐K in primary JEB keratinocytes

Given that PF14 was initially designed for the delivery of oligonucleotides,^33^ we investigated whether the PF14‐K variant could facilitate delivery of short single stranded HDRT for precise gene editing in primary keratinocytes. We used previously published gRNAs and HDRTs designed to correct three pathogenic EB mutations: a splice site mutation in *COL7A1* intron 26 (c.3551‐3T>G),^28^ an 11 bp deletion in *COL7A1* exon 117 (c.8698_8708del),^28^ and a nonsense mutation in *LAMB3* exon 14 (c.1903C>T)^26^ (Figure 5a, Table S3). All repair templates contained at least one silent blocking mutation within the gRNA sequence to prevent recutting (Figure 5a). Additionally, the HDRT targeting *LAMB3* included silent bridging mutations placed between the Cas9 cut site and the mutation site, which we previously demonstrated could enhance the rate of mutation correction.^26^ We first validated this strategy in wild type keratinocytes, using the silent SNPs within each template to assess HDR efficiencies using Sanger sequencing.

**FIGURE 5:**
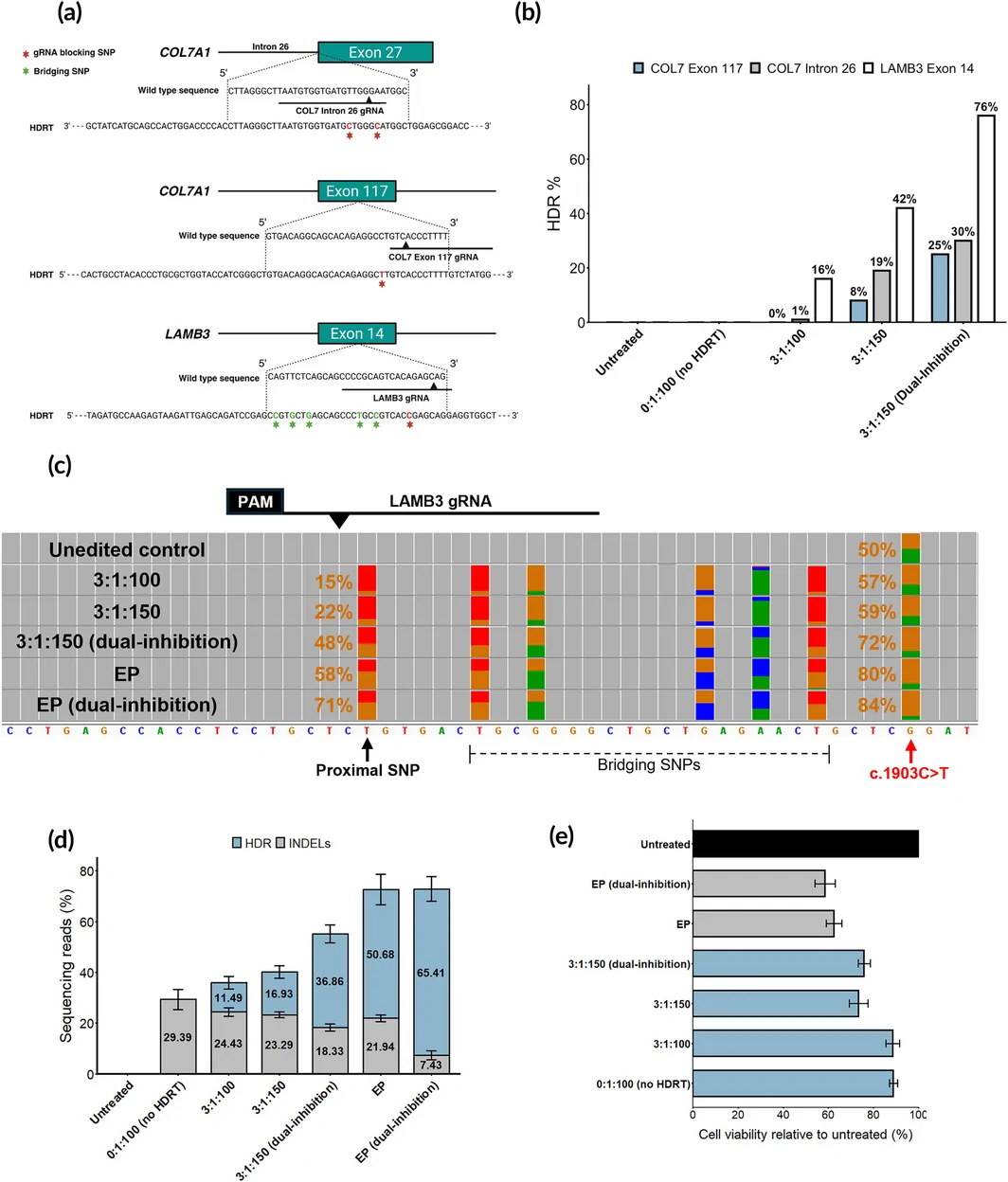
Precise homology‐directed repair (HDR)‐mediated gene editing in primary keratinocytes using short single stranded HDR templates (HDRT) facilitated by PepFect14 variant (PF14‐K). (a) Design of three short single stranded HDRT targeting *COL7A1* or *LAMB3* in primary keratinocytes derived from healthy donors. For each locus, the position of the guide RNA (gRNA) within the wild type sequence is shown, with the Cas9 cut site depicted with an arrow. A portion of the HDRT sequence is depicted below. Blocking mutations to prevent recutting are depicted in red and silent bridging mutations in green, enabling assessment of HDR in wild type cells. (b) HDR efficiencies achieved in wild type primary keratinocytes at all three loci as determined by analysis of Sanger sequencing using the Inference of CRISPR edits online tool. Different HDRT:Cas9‐RNP:PF14‐K molar ratios were assessed. Dual‐inhibition incorporated the small molecule inhibitors ART558 and AZD7648. One biological replicate performed. (c) Raw Nanopore‐based amplicon sequencing data aligned to the reference human genome in interactive genomics viewer displaying a histogram of sequencing read depth following HDR‐mediated gene editing targeting the prevalent c.1903C>T *LAMB3* mutation in primary Junctional EB keratinocytes. The location of the heterozygous mutation is indicated and is present at 50% within the control sequence. The position of the gRNA and the genomic sequence (bottom) is also depicted. Note that the displayed sequence represents the antisense strand (−) in the opposite direction of the LAMB3 HDRT shown in subfigure (A). The proximal SNP closest to the gRNA cut site and its rate of incorporation is shown, along with the bridging mutations positioned between the gRNA cut site and the mutation site. The same conditions were evaluated as in (b). EP, electroporation. (d) Quantification of gene editing efficiencies in primary JEB keratinocytes using CRISPResso2. Bars represent the mean of two biological replicates. Error bars represent the SEM (e) Cell viability analysis of primary JEB keratinocytes following HDR‐mediated gene editing facilitated by PF14‐K or EP. Bars represent the mean of two biological replicates. Errors bars represent the SEM.

The addition of HDRT at a final concentration of 30 nM (determined from preliminary screening, data not shown) resulted in <1% HDR at both *COL7A1* loci and 16% when targeting *LAMB3* (final HDRT:Cas9‐RNP:PF14‐K molar ratio of 3:1:100) (Figure 5b). Increasing the molar excess of PF14‐K (final MR of 3:1:150) led to enhancements in HDR across all three loci (Figure 5b). Higher doses of PF14‐K were not evaluated, as initial screening indicated that they were toxic (Figure 1a). To further enhance efficiencies, we incorporated a dual‐inhibition strategy incorporating the small molecules ART558 and AZD7648 to inhibit the DNA repair enzymes Pol θ and DNA‐PK, respectively. This approach has previously been demonstrated to synergistically enhance precise HDR repair^40^ and similarly led to increases in HDR efficiencies across all three loci (Figure 5b).

Encouraged by the efficient HDR at the *LAMB3* locus, we applied this strategy to primary JEB keratinocytes containing a prevalent heterozygous nonsense mutation (c.1903C>T) which this HDRT was designed to correct.^26^ We employed amplicon‐based Nanopore sequencing to quantify HDR since assessment of Sanger sequencing using the Inference of CRISPR edits (ICE) tool (https://ice.editco.bio/#/) typically overestimates HDR rates. Using a 3:1:100 molar ratio, 15% incorporation of the silent SNP closest to the gRNA cut site (proximal SNP) and 14% correction of the c.1903C>T mutation was achieved, compared to 58% and 60% with electroporation, respectively (Figure 5c). Quantification of the correction of the heterozygous c.1903C>T mutation was calculated based on the increase in the number of sequencing reads harboring a C nucleotide compared to the 50% of sequencing reads (i.e., the wild type allele) in the untreated control. Cas9‐induced DSBs spanning the mutation site were also accounted for and represented as uncorrected alleles (Figure 5c). Increasing the molar ratio of PF14‐K (final molar ratio of 3:1:150) led to enhancements in the incorporation of all silent SNPs, up to 48% for the proximal SNP and 44% mutation correction when using the dual‐inhibition strategy (Figure 5c). Quantification of precise HDR with CRISPResso2 revealed efficiencies of up to 17% with the 3:1:150 molar ratio (Figure 5d). The dual‐inhibition strategy led to an increase in precise mutation correction of up to 37%, while increasing the ratio of precise HDR to total editing events as previously reported.^40^ In comparison, precise mutation correction of up to 51% was achieved with electroporation, increasing to 65% with the dual‐inhibition approach (Figure 5d). However, electroporation was associated with lower cell viability compared to all PF14‐K conditions (Figure 5e). Collectively, these data demonstrate the capacity of PF14‐K to facilitate precise HDR‐mediated repair of primary EB keratinocytes at therapeutic efficiencies and with improved cell viability compared to electroporation.

## 3 DISCUSSION

Safe and efficient delivery of gene editors remains a central challenge of CRISPR‐based gene therapy.^1^, ^3^ Electroporation has served as a primary delivery tool for ex vivo gene editing in preclinical and clinical trials^10^, ^11^ but is hindered by cytotoxicity and limited in vivo applicability.^15^, ^16^ CPPs represent a promising alternative due to their ability to efficiently deliver cargo intracellularly with low toxicity.^16^, ^41^ Since the development of the first CPP—trans‐activator of transcription (TAT) peptide—derived from HIV, many variants have been developed with improved characteristics for facilitating delivery of diverse cargo into a range of cell types and tissues.^17^ Covalent attachment of CPP to RNP has been used to generate deliverable nanoparticles for gene editing.^18^, ^42^, ^43^ However, this method relies on a multistep synthesis and purification process which can complicate manufacturing pipelines and is often inefficient, necessitating large quantities of RNP to facilitate gene editing.^18^, ^42^, ^43^ Additionally, direct conjugation can potentially alter protein function and does not provide a protective shield around the RNP from proteases and RNAses.^44^ Non‐covalently formed RNP‐CPP nanoparticles bypass these limitations and represent a simple strategy requiring only a CPP, which can be commercially sourced at low cost, and RNP. The accessibility of such approaches could encourage wider adoption of CRISPR for fundamental research and potentially offer scalable gene therapy solutions in the future.

Efficient gene editing in this study was facilitated by PF14‐K, an analog that differs from PF14 by a single C‐terminal lysine residue. This variant enabled a ~10% improvement in gene editing in primary human skin cells compared to standard PF14, which Gustafsson et al. previously demonstrated could facilitate Cas9‐RNP gene editing in adherent human cell lines.^21^ Single amino acid changes can substantially impact CPP function by altering various factors including peptide stability, structure and endosome escape.^45^ For example, Porosk et al. revealed that single amino acid substitutions within a PF14 analog substantially altered peptide activity.^45^ This group further demonstrated that substitution of the ornithine residues with lysines on the cationic face of the PF14 α‐helix enhanced miRNA delivery into primary human keratinocytes.^39^ They hypothesized that the increased mobility and hydrophobicity of the longer lysine side chain could enhance peptide interaction with nucleic acids.^39^ Thus, the enhanced gene editing achieved with PF14‐K may result from a stronger interaction with the negatively charged RNP, driven by the lysine residue's longer side chain and additional positive charge within the peptide's cationic face. Capping of short polyalanine peptides with a C‐terminal lysine has been demonstrated to stabilize their α‐helical structure.^46^ Our CD spectroscopic analysis showed that PF14‐K has slightly higher α‐helical content than PF14, providing a potential explanation for the observed improvement in gene editing. Future mechanistic studies should investigate whether this observed increase in gene editing results from improvements in peptide/RNP nanoparticle stability, cellular uptake or endosomal escape. Furthermore, substitution of stearic acid with oleic acid (i.e., PF14‐KO), differing by a single double bond, significantly reduced peptide activity. The saturated structure of stearic acid is thought to facilitate tight packing through self‐associating hydrophobic interactions, stabilizing PF14 nanoparticles and enhancing delivery.^47^ The “kink” introduced by the single double bond of oleic acid may disrupt this packing and compromise nanoparticle stability with Cas9‐RNPs.^37^ However, the mechanistic basis behind these PF14 analogs should be the subject of more comprehensive future research. Collectively, these data highlight the importance of rational design to produce improved PF14 variants for future gene editing applications.

Beyond CPP design, we investigated other factors influencing cellular uptake. Although PF14 contains ornithines for serum stability, we observed improvements in gene editing of up to 15%–20% when using serum‐free conditions. This is consistent with previous data demonstrating that PF14 only retained ~75% of its efficiency in full‐serum medium compared to serum‐free conditions.^33^ Although serum‐free conditions would also be applicable to potential topical applications of PF14‐K in the skin, the presence of skin proteases could potentially influence nanoparticle stability. The solvent used for PF14 has been shown to influence nanoparticle activity.^38^ Biswas et al. demonstrated that using water as a solvent led to the formation of large micelles which could affect nanoparticle activity. They demonstrated that a combination of the polar solvent EtOH and the semi‐polar solvent DMSO was superior for solubilizing both the charged and hydrophobic regions (including the fatty acid component) of amphipathic peptides like PF14.^38^ Additionally, low concentrations of DMSO have been shown to enhance nanoparticle permeability via the formation of hydrophilic pores in the lipid cell membrane.^48^ Using this solvent, we observed subtle but consistent enhancements in gene editing across all tested loci when compared to water, despite these differences not reaching statistical significance. Under optimized conditions with PF14‐K, efficient gene editing of >70%–75% could be consistently achieved in primary dermal fibroblasts, and >50% in primary keratinocytes. Notably, high cell viability of ~90% or higher was obtained with PF14‐K, compared to ~70% with electroporation, enabling similar total edited yields of wild type cells.

We applied our optimized PF14‐K gene editing strategy to target the group of rare genetic skin disorders EB. CRISPR gene editing represents a major goal of EB gene therapy. Preclinical studies have predominantly focused on the generation of skin equivalents composed of ex vivo CRISPR‐edited EB patients cells facilitated by electroporation.^10^ However, consistent with previous studies, electric pulses including electroporation have been shown to alter genes involved in key cellular pathways.^16^, ^49^ A notable result in this study was the observed upregulation of *TGFB1* expression in dermal fibroblasts following electroporation. Urabe and colleagues reported a similar finding in the same cell type following electrical pulse stimulation.^49^ TGF‐β1 is a major driver of RDEB disease progression and contributes to fibroblast activation and differentiation into pro‐inflammatory myofibroblasts, which can promote fibrosis and squamous cell carcinoma.^50^ Thus, the incorporation of myofibroblasts into ex vivo engineered skin equivalents may contribute to the inflammatory environment of RDEB post‐transplantation and influence clinical outcomes. Delivery with CPPs including PF14‐K may ameliorate this effect and better preserve cellular phenotypes. However, only a small panel of genes was assessed in this study. More comprehensive assessment is required to determine the significance of these results.

The gentle nature of PF14‐K was particularly evident in more difficult‐to‐transfect primary EB skin cells. The deletion of faulty *COL7A1* exons is a highly efficient approach demonstrated previously for RDEB using electroporation and has recently gained Orphan drug designation by the EMA.^30^, ^31^, ^32^ In this study, we demonstrated efficient exon 31 deletion and subsequent *COL7A1* reframing at efficiencies of up to 58% in RDEB‐Ks and 68% in RDEB‐Fs as evidenced by amplicon‐based Nanopore sequencing. While PCR bias could potentially inflate exon deletion efficiencies by preferentially amplifying the shorter, exon deleted amplicon, flow cytometric analysis of RDEB‐Ks following dual Cas9‐RNP exon skipping revealed COL7 expression at comparable levels to the editing efficiencies observed at the genomic level. Notably, improved cell viability was observed with peptide‐edited cells, enabling similar total edited cell yields compared to electroporation. Gene editing efficiencies of 20%–35% have been reported necessary for therapeutic use in an EB context.^10^ Thus, provided these efficiencies can be achieved, bulk edited cells obtained using peptide‐mediated delivery may provide a better therapeutic product for clinical use since cell viability and the transcriptome^16^ are better maintained. The electroporation process has also been shown to induce DNA damage, resulting in potentially genotoxic events,^15^ however future research should compare this with CPP‐mediated delivery. Further, PF14‐K precisely corrected a prevalent *LAMB3* mutation for JEB at efficiencies of up to 37%. However, this was achieved in the presence of the DNA repair inhibitor AZD7648 which has been shown to cause large‐scale genomic alterations.^51^ Although the dual‐inhibition strategy we employed has been demonstrated to mitigate off‐target genotoxicity due to the inclusion of a Polϴ inhibitor,^40^ thorough genome‐wide off‐target analysis should be conducted in the future to determine the safety of this approach. Additionally, 3D skin models should be considered in the future to determine the efficacy of PF14‐K to mediate in vivo delivery into the skin. To the best of our knowledge, this is the first example of HDR‐mediated repair or exon skipping targeting a pathogenic mutation in primary human cells facilitated by a CPP. Similar HDR‐mediated repair strategies have been performed with other CPPs; however they have typically been performed in cell lines which are highly permissible to transfection, targeting reporter constructs or safe harbor loci.^20^, ^21^, ^22^, ^23^

Collectively, these data establish PF14‐K as a gentler alternative delivery method to electroporation in primary human skin cells. PF14‐K has been shown to enter cells through class A scavenger receptors,^52^ making it a potentially effective candidate for different cell types with high scavenger receptor expression. Beyond HDR, this strategy has the potential to work with prime and base editors. For example, Gustafsson et al. employed a mix‐and‐incubate strategy with the hPep3 amphipathic CPP to deliver base editors into iPSCs and muscle stem cells, achieving efficiencies of up to 80%, further highlighting the potential of CPPs to facilitate efficient gene editing in difficult‐to‐transfect human cells.^41^ Additionally, delivery of base and prime editors bypasses the need to deliver HDRT which suffer from inefficient nuclear delivery. PF14 was previously used to deliver miRNA via subcutaneous injection into a mouse model of irritant contact dermatitis to alleviate inflammation.^39^ Therefore the capacity of PF14‐K to deliver gene editors into the skin could be investigated in the future for EB.

## 4 MATERIALS AND METHODS

### 4.1 Primary cell culture

Primary keratinocytes and neonatal dermal fibroblasts were purchased from the American Type Culture Collection (ATCC). Patient‐derived primary skin cells were isolated from fully informed and consenting patients with JEB and RDEB (ethical approval number 19/STH/47 as provided by the Southern Health and Disability Ethics Committee) by whole skin digest of skin biopsies as described previously.^53^

Primary keratinocytes were cultured on a feeder layer of lethally irradiated 3T3‐J2 murine fibroblasts (Kerafast) in modified Kelch's medium as described previously^26^ (DMEM [no calcium chloride]:F12 3:1 [Gibco], 10% FBS [Moregate], 100 U/mL penicillin/streptomycin [Gibco], 0.625 μg/mL amphotericin B [Sigma‐Aldrich], 0.4 μmol/L SB 772077B ROCK inhibitor [Tocris] and 20 ng/mL KGF [Peprotech, Rocky Hill, NJ]). Primary dermal fibroblasts were cultured as described previously^26^ in DF10 (DMEM [Gibco], 10% FBS [Moregate] and 100 U/mL penicillin/streptomycin [Gibco]).

### 4.2 Peptides

Peptides were synthesized using Fmoc‐solid‐phase peptide synthesis (Fmoc‐SPSS) (>98% purity) and stored lyophilized or as 1 mM stocks in water or 90% EtOH and 10% DMSO at −80°C. Peptide sequences are shown in Figure 3a.

### 4.3 Small molecule inhibition of DNA repair

AZD7648 and ART558 (MedChemExpress) were reconstituted in DMSO as 20 mM or 10 mM base stocks, respectively, and stored at −80°C. For use in HDR‐mediated gene editing experiments, AZD7648 and ART558 were added to the cell culture medium immediately prior to transfection at a final concentration of 1 μM or 5 μM, as indicated. A complete medium change was performed after 24 h to remove the inhibitors.

### 4.4 RNP and peptide complex formation and delivery

For peptide‐mediated delivery experiments, cells were seeded at 20,000/cm^2^ in a 96‐ or 48‐well plate and cultured for 24 h at 37°C. The following day, RNPs were formed using a 2.5:1 molar ratio of Alt‐R™ sgRNA (IDT): high‐fidelity Alt‐R™ S.p. Cas9 Nuclease V3 (IDT) (sgRNA sequences are shown in Table S1). Cas9 nuclease and sgRNAs were always diluted in PBS before RNP formation as this resulted in higher gene editing rates. For HDR‐mediated gene editing experiments, varying molar ratios of single‐stranded HDR repair templates (HDRTs, described in‐text) were added to preformed RNPs and incubated for 1 min (HDRT sequences are shown in Table S3). One millimolar peptide stocks (reconstituted in water or 90% EtOH and 10% DMSO) were then complexed at varying molar ratios with RNPs (with or without HDRT) as indicated in‐text in complexation buffer (1:1 v/v ratio of 5% w/v PEG‐PVA [with 6.3% w/v sucrose] and HBG buffer [20 mM HEPEs, 5% w/v glucose, pH 7.2]) for 40 min at room temperature, with the concentration of RNP (as defined by the Cas9 concentration) kept constant at 22.86 ng/μL. Complexation was performed in low‐binding PCR tubes. The final complexes were added to the medium of cells in culture, with the final treatment volume equating to 7% of the total volume (for example, 35 μL in 500 μL total volume). Unless stated otherwise, the final optimized dose of RNP (as defined by Cas9) was 800 ng per well in a 48‐well plate or 160 ng per well in a 96‐well plate at a final concentration of 10 nM, with 100 molar equivalents of peptide (1 μM final peptide concentration). A vehicle control containing the same volume of complexation buffer was included in all experiments. After 24 h a complete medium change was performed to remove the treatment. For serum‐free peptide experiments, Kelch's medium or DF10 without FBS was used, and 10% FBS was added back to the cell culture medium during the medium change.

### 4.5 RNP electroporation

A 4D nucleofector (Lonza) was used for electroporation of primary skin cells. RNPs were formed using a 4.76:1 molar ratio of sgRNA: high‐fidelity Cas9 (75 pmol sgRNA and 15.75 pmol Cas9) and subsequently electroporated into 1 × 10^5^ primary dermal fibroblasts or keratinocytes using the P2 primary cell kit (Lonza) with the pulse code DT‐130 or the P3 primary cell kit (Lonza) with the pulse program CM‐137, respectively. For HDR‐mediated gene editing experiments, 50 pmol of single stranded HDRT was incubated with preformed RNPs for 1 min prior to electroporation. Following electroporation, the cells were rested for 10 min at 37°C and then seeded into cell culture plates containing prewarmed culture medium with DNA repair inhibitors as indicated.

### 4.6 On‐target genotyping

Cells were pelleted by centrifugation and resuspended in 100 μL of cold DPBS containing 5 μL of Liquid Proteinase K (Machery‐Nagel) and gDNA extracted using the Monarch® gDNA extraction kit (NEB) following the manufacturer's instructions. gDNA was used as an input for PCR using Phusion Hot Start II DNA Polymerase using thermal cycle settings as described previously^31^ (primers are listed in Table S2). PCR amplicons were genotyped using Sanger sequencing or amplicon‐based Nanopore sequencing as described below.

### 4.7 Amplicon‐based Oxford nanopore sequencing

Nanopore sequencing libraries were prepared using the Oxford Nanopore Rapid Barcoding Kit 24V14 (SQK‐RBK114.24) following the manufacturer's guidelines. Prepared libraries were loaded onto MinION flow cells (FLO‐MIN114) and sequenced on a MinION sequencer (ONT, Mk1B). Fastq files were generated using Guppy basecaller and then aligned to the human reference genome (GRCh38) and filtered for reads spanning the target site with minimap2 (version 2.24) and SAMtools (version 1.15). The cleaned fastq files were used to generate Binary alignment map (BAM) files which were visualized in interactive genomics viewer (IGV). For HDR‐mediated gene editing experiments, processed fastq files were analyzed using CRISPResso2 (https://github.com/pinellolab/CRISPResso2). CRISPRlungo (https://pinellolab.github.io/CRISPRLungo/) was used for analyzing exon deletion efficiencies.^54^

### 4.8 Electrophoretic mobility shift assay

Peptide/RNP complex formation was assessed using an electrophoretic mobility shift assay on a 2% agarose gel in TAE buffer (pH 8.3) containing the nucleic acid binding dye SYBR Safe (Invitrogen). RNPs were formed using 1 μg of sgRNA in a 2:1 sgRNA:Cas9 molar ratio and then complexed with peptide at varying molar ratios in HBG buffer (20 mM HEPEs, 5% w/v glucose, pH 7.2) for 40 min at room temperature. RNP and sgRNA only controls were included. Samples were run for 30 min at 110 V and the gels imaged with a Bio‐Rad Gel Doc XR+ imager. Gels were visualized with Image Lab™ software.

### 4.9 Alamar blue cell viability assay

Cell viability was assessed using the AlamarBlue™ high sensitivity cell viability reagent (Invitrogen) following the manufacturer's guidelines. Absorbance was measured with a SpectraMax iD3 multi‐mode spectrophotometer.

### 4.10 RT‐PCR and droplet digital PCR

ddPCR was used to quantify gene expression following peptide or electroporation‐mediated gene editing. Peptide‐mediated gene editing was scaled up to a 12‐well plate to ensure sufficient cell numbers for RNA extraction and performed as described above. Cells were seeded at 20,000/cm^2^ and cultured for 24 h before peptide treatment using an optimized RNP concentration of 10 nM at a 1:100 molar ratio of RNP to peptide. RNP electroporation samples were prepared as described above. Twenty‐four hours after gene editing total RNA was extracted using the RNAqueous Total RNA Isolation Kit (Thermo Fisher Scientific) and cDNA synthesis performed with the SuperScript III First‐Strand Synthesis kit (Invitrogen) following the manufacturer's instructions. The QX200 ddPCR system (Bio‐Rad) was used for gene expression quantification using the EvaGreen binding dye. Primers are listed in Table S2.

### 4.11 Flow cytometry

COL7 expression in keratinocytes was detected via flow cytometry. For each sample, approximately 2–5 × 10^5^ cells were labeled using the Zombie NIR Fixable Viability Kit (Biolegend, San Deigo, CA, USA) and then fixed and permeabilized using the eBioscience Foxp3/transcription factor staining buffer set (Thermo Fisher Scientific) according to manufacturer's instructions. Samples were washed with permeabilization buffer and resuspended in permeabilization buffer supplemented with 4% v/v goat serum (Gibco) for 60 min RT then stained with 0.1 μg/mL anti‐human‐COL7 clone LH7.2 (Invitrogen) overnight at 4°C. LH7.2 was removed by washing with permeabilization buffer and samples were then incubated in permeabilization buffer supplemented with 1 mg/mL polyclonal goat anti‐mouse IgG1‐AlexaFluor647 (Invitrogen) at RT for 60 min. Unstained cells, cells labeled only with Zombie NIR, and cells labeled with Zombie NIR and incubated with secondary antibody in the absence of LH7.2 were used as negative and gating controls. Keratinocytes from healthy donors were included as positive expression controls for COL7. Samples were acquired on a Cytek Northern Lights flow cytometer and analyzed using FlowJo Software vX (BD Biosciences). Flow cytometry acquisition was performed at the Auckland Cytometry Shared Research Equipment Centre, School of Biological Sciences, University of Auckland.

### 4.12 Immunofluorescence

Delivery of Cas9‐RNP/peptide complexes into primary human fibroblasts was visualized using a red‐fluorescent protein tagged Cas9 (RFP‐Cas9) (IDT). One thousand five hundred cells were seeded into a 96‐well Optical‐Bottom Microplate (Nunc) and cultured for 24 h. RNPs were formed using a 2.5:1 molar ratio of RFP‐Cas9 and sgRNA for 10 min, and a 100 molar excess of peptide complexed with the preformed RNPs in complexation buffer as described above. Cells were treated with RNP only or RNP/peptide complexes and subsequently fixed after 1, 24, or 48 h with 4% paraformaldehyde for 15 min at room temperature, followed by permeabilization with 0.5% Triton X‐100 for a further 15 min at room temperature. Following a 10‐min PBS wash, cells were stained with DAPI and Alexa Fluor™ 488‐labeled phalloidin in dilution buffer (10% human serum in PBS) for 1 h at room temperature. Samples were then washed two times with PBS for 15 min each. Confocal z‐stacks were acquired using a Nikon Eclipse Ji Microscope equipped with a water‐immersion lens, with identical exposure settings applied across all samples. Image analysis was performed in ImageJ.

### 4.13 Transmission electron microscopy

Visualization of Cas9‐RNP/PF14 nanoparticles using TEM was performed using negative uranyl acetate staining. Cas9‐RNP and PF14 were complexed as described above for 40 min at a 1:100 molar ratio in HBG buffer. A sample with RNP but without PF14 was included as a negative control. Undiluted samples were adsorbed for 1 min to freshly glow‐discharged carbon formvar grids (copper, 400‐mesh, Electron Microscopy Sciences, Morgantown, PA, USA). Grids were washed briefly with MilliQ water, then negative stained for 30 s with 2% (w/v) aqueous uranyl acetate and imaged using a Tecnai F20 TEM (Thermo Fisher, Eindhovem, The Netherlands) operating at 200 kV, with a TemCam‐XF416(ES) CMOS detector (TVIPS, Gilching, Germany).

### 4.14 Circular dichroism spectroscopy

Secondary structure of PF14 analogs was assessed using CD spectroscopy. Concentrations of peptide stock solutions were calculated from absorbance measurements at 280 nm, assuming an absorption coefficient of 1490 M^−1^ cm^−1^. Stock solutions of peptides were then diluted in 10 mM NaP buffer (pH 7.2) to a final concentration of 25 μM. Spectra were recorded using a Chirascan spectrophotometer (Applied Photophysics) with an optical bandwidth of 1 nm and a spectral step size of 0.5 nm. Spectra were smoothed by averaging over a 5 point (2.5 nm) moving window, and the final baseline‐corrected spectra reported in terms of the CD absorption coefficient per mol of peptide bond Δ*ε* (M^−1^ cm^−1^) = Δ*A*/(*c* [M] × *l* [cm]), where Δ*A* is the differential absorbance of left and right circularly polarized light, c is the molar concentration of peptide bonds, and *l* is the optical path length.

### 4.15 Dynamic light scattering

Hydrodynamic diameter and PDI of Cas9‐RNP/peptide nanoparticles were measured using a Malvern Zetasizer Advanced Pro with ZS Xplorer software. Peptide and Cas9‐RNPs were complexed as described above in 10 mM HEPEs buffer (pH 7.2). Five measurements were taken per run at 20°C.

### 4.16 Statistical analysis

Statistical tests were performed using RStudio to determine statistically significant differences between samples, with the specific tests described in the relevant figure captions. *p < 0.05, **p < 0.01, ***p < 0.001. Data are represented as the mean ± SEM.

## AUTHOR CONTRIBUTIONS

**Alex du Rand**, **Courtney Masterson**, and **Daniel Verdon** designed and performed cellular and molecular experiments. **Evert Loef** conducted immunofluorescent imaging. **Andrew Siow** synthesized peptides. **Alex du Rand** and **Courtney Masterson** performed data analysis. **Rod Dunbar** and **Paul Harris** provided resource support. **Hilary Sheppard** conceived the topic of the manuscript, supervised the project, and provided resource support. **Alex du Rand** wrote the manuscript. **Richard Kingston** provided support for CD spectroscopy. All authors critically reviewed the manuscript.

## FUNDING INFORMATION

This work was supported by grants from the Auckland Medical Research Foundation (AMRF) and the Maurice Wilkins Centre. Marsden Fund, grant number 23‐UOA‐231.

## CONFLICT OF INTEREST STATEMENT

The authors declare no competing interests.

## Supporting information


**Figure S1.** Effect of complexation buffer on gene editing facilitated by PF14/Cas9‐RNP nanoparticles in primary human dermal fibroblasts. Gene editing efficiencies across two gRNA sites facilitated by PF14 and Cas9‐RNPs complexed in either a 1:1 ratio of polyethylene glycol‐polyvinyl alcohol (PEG‐PVA) buffer and HEPEs buffered glucose (HBG), or HBG only. Bars represent the mean of three biological replicates. Error bars represent the SEM Individual datapoints are represented as shapes from matched experiments. Significant differences were determined using a Welch's *t*‐test with Holm‐Šídák correction for multiple comparisons. ** < 0.01.

**Figure S2.** Cell viability of primary human skin cells following CRISPR gene editing facilitated by PF14‐K/Cas9‐RNP nanoparticles or electroporation. Cell viability was measured 24 h after electroporation (EP) or PF14‐K treatment across four gRNA sites in primary human fibroblasts (A) or primary human keratinocytes (B). Bars represent the mean of two biological replicates. Error bars represent the SEM (C) Total live cell numbers between PF14‐K‐treated primary skin cells compared to an untreated control 1 week after peptide‐mediated delivery. Bars represent the mean of two biological replicates. Error bars represent the SEM.

**Figure S3.** CD spectra of PF14 and PF14‐K at varying temperature. Spectra at the indicated temperatures are reported in terms of the differential molar absorption coefficient (Δ*ε*) per mol of peptide bond.

**Figure S4.** Cell viability of RDEB keratinocytes following dual Cas9‐RNP exon skipping targeting exon *COL7A1* exon 31 facilitated by PF14‐K or electroporation. Viability was measured 24 h after treatment. Bars represent the mean of three biological replicates. Error bars represent the SEM Significant differences were determined using a Welch's *t*‐test with Holm‐Šídák correction for multiple comparisons. ** < 0.01.

**Figure S5.** Flow cytometry scatterplots illustrating representative gating strategies for the detection of COL7. Left to right: All cells; single cells; live cells; (zombie‐NIR viability stain); COL7‐positive cells (Alexa Fluor 647). Percentages within each gate are indicated in red. Gating strategy is depicted for (A) secondary‐only control and (B) anti‐COL7 and secondary antibody staining using paired primary RDEB keratinocyte samples following dual Cas9‐RNP‐mediated restoration of COL7 expression.

**Figure S6.** Flow cytometry scatterplots illustrating COL7 restoration following dual Cas9‐RNP gene editing facilitated by PF14‐K compared to electroporation in primary RDEB keratinocytes. Three biological replicates depicted for PF14‐K‐mediated gene editing (top) compared to electroporated (EP) RDEB keratinocytes, untreated RDEB keratinocytes, and normal human keratinocytes (NHK).

**Table S1.** List of guide RNA (gRNA) sequences. gRNA name (as written in‐text) and the genomic target are shown (based on the *COL7A1* reference transcript, accession number NM_000094.3). PAM sites are indicated in red.
**Table S2**. List of primer sequences.
**Table S3**. List of HDR templates.

## Data Availability

The data from this study are available from the corresponding author on reasonable request.
