# Supplementary material for: A PepFect14 analog improves non‐viral CRISPR delivery in primary human cells to facilitate genome editing and repair
Source: Bioeng Transl Med. 2026 Aug 28;11(6):e70172. doi: 10.1002/btm2.70172 (PMC13522898; doi:10.1002/btm2.70172)
Supplement: Supplementary file 1 [file BTM2-11-e70172-s004.docx]

**A Novel PepFect14 Analog Improves Non-Viral CRISPR Delivery in Primary Human Cells to Facilitate Genome Editing and Repair**

**The Journal of Bioengineering & Translational Medicine**

Alex du Rand, Courtney Masterson, Daniel Verdon, Andrew Siow, Evert Loef, Rod Dunbar, Richard Kingston, Paul Harris, Hilary Sheppard

Corresponding author: Hilary Sheppard ([h.sheppard@auckland.ac.nz](mailto:h.sheppard@auckland.ac.nz)), the School of Biological Sciences, the University of Auckland, Auckland, New Zealand.

| **Name** | **Target** | **Sequence (5’-3’)** |
| --- | --- | --- |
| COL7 intron 31 | *COL7A1*, intron 30 | UAGGUGGGGAUAAGCCAGUC-AGG |
| COL7 intron 32 | *COL7A1*, intron 31 | GAUAAGCCAGUCAGGGUGCA-GGG |
| COL7 intron 67 | *COL7A1*, intron 67 | CUUUGUGUUCUGCCGUAUCU-GGG |
| COL7 intron 68 | *COL7A1*, intron 68 | CCAAGGUGAGUGCGGAUGUU-GGG |
| COL7 intron 108 | *COL7A1*, intron 108 | CAUUAGGCCAGGGAGCUCAU-GGG |
| COL7 intron 109 | *COL7A1*, intron 109 | AUUGAGGGGAGCGUGCACUU-TGG |
| LAMB3 | *LAMB3*, exon 14 | CCCCGCAGUCACAGAGCAGG-AGG |
| COL7 intron 26 | *COL7A1*, intron 26/exon 27 border | TAATGTGGTGATGTTGGGAA-TGG |
| COL7 exon 117 | *COL7A1,* exon 117 | ACCATAGACAAAAGGGTGAC-AGG |

**Table S1. List of guide RNA (gRNA) sequences**. gRNA name (as written in-text) and the genomic target are shown (based on the *COL7A1* reference transcript, accession number NM_000094.3). PAM sites are indicated in red.

| **Target** | **Analysis** | **Sequence (5’-3’)** |
| --- | --- | --- |
| *COL7A1*, Intron 26/Exon 27 | On-target genotyping | **FWD:** TCCTCCTCAGGCTCTGTGTT  **REV:** TCACCATCTCTCCAGGTTCC |
| *COL7A1*, Exon 31 | On-target genotyping | **FWD**: CCTGTGTCAGGCATCCTTCA  **REV**: GTCCCCCAAAGTCTCTGTGG |
| *COL7A1*, Exon 68 | On-target genotyping | **FWD**: GCAGGCACACCCTTAGACAT  **REV**: TTGCCCAGAGCACCCTTTAG |
| *COL7A1*, Exon 109 | On-target genotyping | **FWD**: GGTGTAGGGACAAGGAAGGC  **REV**: GTCAGTGCAGCTTCTCCCTT |
| *COL7A1*, Exon 117 | On-target genotyping | **FWD:** CCCCCTGAGGATGATGAGTA  **REV:** CACACAAGCCTCTAGCACCA |
| *LAMB3*, Exon 14 | On-target genotyping | **FWD:** GCGAGGCTACTGTAATCGCT  **REV:** CGGGACCAACTTCCATTCCA |
| *COL7A1* cDNA (Exon 109) | ddPCR | **FWD**: GTGACCAGGGCGAGAAA  **REV**: CAGCAGAGCCATCATTTCCA |
| *HPRT-1* cDNA | ddPCR | **FWD**: GCTGAGGATTTGGAAAGGGT  **REV**: CCTTCATCACATCTCGAGCAA |
| *TBP* cDNA | ddPCR | **FWD**: GAGTTCTGGGATTGTACCGC  **REV**: CACGAAGTGCAATGGTCTTT |

**Table S2. List of primer sequences**.

| **Name** | **Sequence (5’-3’)** |
| --- | --- |
| COL7 Intron 26 | GGCTGATGGGGAAGGGGTCTGGGAATAGGGGTGGCCCTGAAAAGGCTATCATGCAGCCACTGGACCCCACCTTAGGGCTTAATGTGGTGATGCTGGGCATGGCTGGAGCGGACCCAGAGCAGCTGCGTCGCTTGGCGCCGGGTAT |
| COL7 Exon 117 | TGGATGAGGGCTCCTGCACTGCCTACACCCTGCGCTGGTACCATCGGGCTGTGACAGGCAGCACAGAGGCTTGTCACCCTT TTGTCTATGGTGGCTGTGGAGGGAATGCCA |
| LAMB3 Exon 14 | GGCTGGAGGACCGTGGCCTGGCCTCCCGGATCCTAGATGCCAAGAGTAAGATTGAGCAGATCCGAGCCGTGCTGAGCAGCCCTGCCGTCACCGAGCAGGAGGTGGCTCAGGTGGCCAGTGCCATCCTCTCCC |

**Table S3. List of HDR templates.**
